# Parental participation in the care of hospitalized neonates in low- and middle-income countries: A systematic review and meta-analysis

**DOI:** 10.3389/fped.2022.987228

**Published:** 2022-08-25

**Authors:** Anna Reiter, Julie De Meulemeester, Nathan Kenya-Mugisha, Abner Tagoola, Olive Kabajaasi, Matthew O. Wiens, Jessica Duby

**Affiliations:** ^1^Faculty of Medicine and Health Sciences, McGill University, Montreal, QC, Canada; ^2^Walimu, Kampala, Uganda; ^3^Jinja Regional Referral Hospital, Jinja, Uganda; ^4^Center for International Child Health, BC Children’s Hospital, Vancouver, BC, Canada; ^5^Department of Anesthesiology, Pharmacology & Therapeutics, Faculty of Medicine, University of British Columbia (UBC), Vancouver, BC, Canada; ^6^Department of Pediatrics, McGill University, Montreal, QC, Canada

**Keywords:** infant, nursing, patient-centered care, family nursing, parents

## Abstract

**Introduction:**

To determine the effect of parental participation in hospital care on neonatal and parental outcomes in low- and middle-income countries (LMICs) and to identify the range of parental duties in the care of hospitalized neonates in LMICs.

**Methods:**

We searched CINAHL, CENTRAL, LILACs, MEDLINE, EMBASE and Web of Science from inception to February 2022. Randomized and non-randomized studies from LMICs were eligible if parents performed one or more roles traditionally undertaken by healthcare staff. The primary outcome was hospital length-of-stay. Secondary outcomes included mortality, readmission, breastfeeding, growth, development and parental well-being. Data was extracted in duplicate by two independent reviewers using a piloted extraction form.

**Results:**

Eighteen studies (eight randomized and ten non-randomized) were included from seven middle-income countries. The types of parental participation included hygiene and infection prevention, feeding, monitoring and documentation, respiratory care, developmental care, medication administration and decision making. Meta-analyses showed that parental participation was not associated with hospital length-of-stay (MD −2.35, 95% CI −6.78–2.07). However, parental involvement was associated with decreased mortality (OR 0.46, 95% CI 0.22–0.95), increased breastfeeding (OR 2.97 95% CI 1.65–5.35) and decreased hospital readmission (OR 0.36, 95% CI 0.16–0.81). Narrative synthesis demonstrated additional benefits for growth, short-term neurodevelopment and parental well-being. Ten of the eighteen studies had a high risk of bias.

**Conclusion:**

Parental participation in neonatal hospital care is associated with improvement in several key neonatal outcomes in middle-income countries. The lack of data from low-income countries suggests that there remains barriers to parental participation in resource-poor settings.

**Systematic review registration:**

[https://www.crd.york.ac.uk/prospero/display_record.php?RecordID=187562], identifier [CRD42020187562].

## Introduction

Despite improved coverage of facility-based maternal and neonatal care over the past two decades, the neonatal mortality rate in low- and middle-income countries (LMICs) remains high (1). Sub-optimal quality of care in hospitals contributes to the persistence of preventable neonatal deaths in LMICs. In 2016, the World Health Organization (WHO) published standards for improving the quality of maternal and neonatal care in healthcare facilities (2). However, the shortage of healthcare workers in LMICs is a significant barrier to achieving these quality standards. In 2018, 60% of the least developed countries failed to meet the WHO minimum threshold of 3 nurses or midwives per 1,000 people (3).

In LMICs, parents, usually mothers, remain at the bedside of their hospitalized child and become the *de facto* primary caretakers when there is a shortage of medical personnel. Hence, parents may be uniquely positioned to improve the quality of hospital-based care with proper training. For example, parental involvement in Kangaroo Mother Care (KMC) has led to significant reductions in mortality and morbidities for hospitalized neonates in LMICs (4). However, it remains unknown whether parents can assume more complex responsibilities typically assigned to nursing or medical personnel in LMICs.

Therefore, the primary objective of this systematic review was to identify studies that evaluated the impact of parental participation in hospital care on neonatal and parental outcomes in LMICs. We also sought to identify the range of parental duties evaluated by the eligible studies in the care of hospitalized neonates in LMICs.

## Methods

This systematic review was registered on the International Prospective Register of Systematic Reviews (CRD42020187562) and conducted in accordance with the Preferred Reporting Items for Systematic Reviews and Meta-Analyses (PRISMA) statement (5).

### Eligibility

Studies comparing parental participation in the care of their hospitalized neonates to standard nursing care were included. Parental participation was defined as the parental assignment of duties that would traditionally be performed by the inpatient nursing or medical team. Such interventions included but were not limited to bathing, vital sign monitoring, bottle feeding, diaper changing, hand hygiene and infection control measures, identification of signs of deterioration, measuring growth and resuscitation. Studies that evaluated forms of parental participation that would not otherwise be provided by the nursing or medical team, such as Kangaroo Mother Care, breastfeeding, or family participation in rounds, were excluded unless they were part of a larger intervention that also included participation in nursing or medical care.

Randomized and non-randomized comparative studies were eligible for inclusion if they occurred in LMICs as defined by the World Bank in 2022 (6). Hospitalized neonates were defined as patients with a corrected gestational age < 44 weeks at the time of enrollment, irrespective of gestational age, birthweight, or reason for hospital admission.

We chose the primary outcome of hospital length-of-stay because enhanced parental involvement has been shown to decrease length-of-stay in neonatal intensive care units in high-income countries (7, 8). We also examined a range of secondary outcomes related to the neonate (mortality, growth, breastfeeding, nosocomial infection, readmission, and neurodevelopment), the parent (satisfaction, perceptions of participation, coping, discharge preparedness) and the healthcare system (cost).

### Study selection and data extraction

We searched CINAHL, CENTRAL, LILACs, MEDLINE, EMBASE and Web of Science from inception to February 27, 2022. The search strategy was developed in part by using search terms from the Cochrane Neonatal Standard Search Strategy and the Cochrane LMIC filter (Supplementary Material).

Two reviewers (JDM and AR) independently screened all titles and abstracts for relevance. The full texts of all relevant and potentially relevant studies were retrieved, and two reviewers (JDM and AR) independently determined the eligibility of retrieved studies using predefined eligibility forms. Disagreements were resolved through discussion with the assistance of a senior reviewer (JD). We used EndNote 20 for reference management, and Covidence systematic review software (Veritas Health Innovation, Melbourne, Australia) for data management.

Two reviewers (JDM and AR) independently extracted data in duplicate from all eligible studies using a piloted data extraction form. The following data items were extracted: title; author and year of publication; eligibility criteria; study aim; study design; study duration; country; hospital setting description; population description; number of participants; intervention characteristics, including description, duration, providers, and resource requirements; primary and secondary outcome measures; and conclusions. Study authors were contacted via email if clarification was required.

### Analysis

We performed meta-analyses to estimate the treatment effect if at least three studies evaluated the outcome of interest. We also performed secondary analyses restricted to randomized controlled trials if at least two randomized controlled trials were included. We calculated summary odds ratios (ORs) for dichotomous data and mean differences (MDs) for continuous variables, both with 95% confidence intervals (CI). Pooled estimates were obtained through random-effect models due to the varied nature of the interventions included. Heterogeneity between studies was measured using the *I*^2^ statistic. We used Review Manager V. 5.4.1 to conduct the meta-analyses. When meta-analysis was not feasible, we performed a narrative synthesis. To assess risk-of-bias, we used the Cochrane RoB 2 for randomized studies (9) and ROBINS-I for non-randomized studies (10). Two independent reviewers (JDM and AR) assessed risk of bias in duplicate and disagreements were resolved through discussion with a senior reviewer (JD).

## Results

**FIGURE 1:**
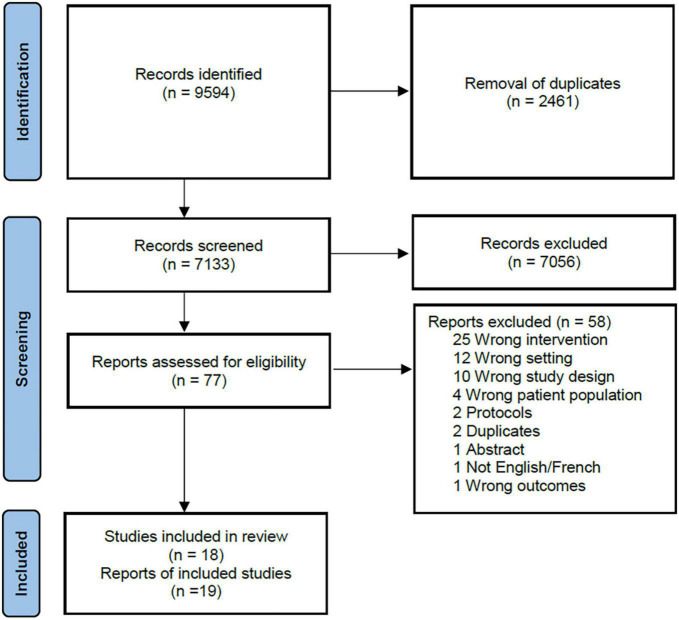
PRISMA flow diagram.

Of the 7,133 abstracts that were screened, 77 full-text articles were assessed. A total of 19 reports (representing 18 studies with one study publishing results in two unique reports) met our inclusion criteria and were included in the analysis (Figure 1). Eight (44.4%) were randomized controlled trials (11–19), two (11.1%) were quasi-experimental studies (20, 21), one (5.6%) was a retrospective cohort study (22), five (27.8%) were pre-post intervention studies (23–27), and two 11.1%) were non-randomized controlled trials (28, 29) (Supplementary Table 1). Studies were conducted in upper-middle income countries (12–17, 19–22, 24, 25, 28) or lower-middle income countries (11, 23, 26, 27, 29) with no representation from low-income countries.

We classified the individual caretaking responsibilities into seven main domains: hygiene and infection prevention (*n* = 10, 55.6%) (12, 14, 15, 18, 19, 21, 23, 24, 26, 27), feeding (*n* = 18, 100.0%), monitoring and documentation (*n* = 9, 50.0%) (11, 14–18, 21–23, 27) respiratory care (*n* = 2, 11.1%) (19, 23), developmental care (*n* = 14, 77.8%) (11–20, 24–26, 28, 29); medication administration (*n* = 2, 11.1%) (15, 22), and decision making (*n* = 3, 16.7%) (20, 22, 24). All but two studies (20, 29) described the parental training required prior to parental involvement. Nursing staff provided the training in most studies in which the profession of the trainers was described (18, 21, 22, 24–26), but the research teams (12, 16–18, 28) and other members of the healthcare team (18) were also involved in select studies. A variety of formats, such as video lectures, pamphlets, or hands-on sessions were used to teach the parents the necessary skills. When described, the duration of the training sessions ranged from a single 30–45 minute session (12) to daily teaching for two hours a day for the length of hospitalization (18). No studies directly described if and/or how parents were monitored to ensure that they completed their assigned caretaking responsibilities in the intervention group. However, one study (16) reports poor compliance as a reason for exclusion for study participants.

In seven studies (38.9%), parents in the control group had minimal to no interaction with their child during hospitalization (12, 19–22, 24, 26). In eight studies (44.4%) (11, 13, 15–18, 23, 27, 29), parents in the control group were encouraged to visit their child regularly and assumed traditional parental responsibilities, including breastfeeding and/or Kangaroo mother care. Three studies did not specify the nature of the standard care provided to the control group (14, 25, 28). For the intervention group, parental participation was restricted to the neonate’s mother (or other female guardian) in all but seven studies (18–22, 24, 28).

### Hospital length of stay

**FIGURE 2:**
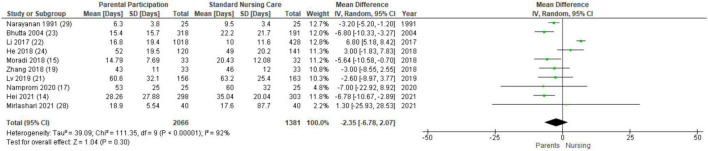
Forest plot and meta-analysis of the association between parental participation and length of stay.

Thirteen studies evaluated length of stay (14, 15, 17–24, 26, 28, 29). The overall duration of hospitalization across studies varied widely with averages for both the intervention and control groups ranging from less than 10 days in two studies (26, 29) to more than 40 days in four trials (17, 19, 21, 24). Ten studies provided data that was conducive for a meta-analysis (14, 15, 17, 19, 21–24, 28, 29). Parental participation did not affect length of stay when compared to standard nursing care (MD −2.35, 95% CI −6.78–2.07, *I*^2^ = 92%; Figure 2), and the result remained similar when restricted to randomized controlled trials (MD 1.16, 95% CI −14.6–16.93). In the three studies not included in the meta-analysis, length of stay was reported to be similar between the intervention and control groups (18, 20, 26).

### Mortality

**FIGURE 3:**
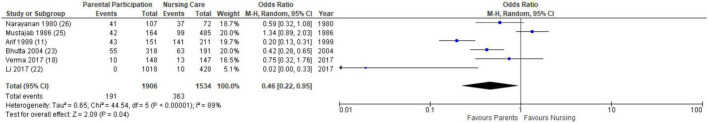
Forest plot and meta-analysis of the association between parental participation and mortality.

Seven studies studied neonatal mortality during hospitalization (11, 18, 22, 23, 25–27), and six of these studies were included in a meta-analysis (11, 18, 22, 23, 25, 26). Compared to neonates who received standard nursing care, neonates whose parents participated in hospital duties were less likely to die during hospitalization (OR 0.46 95% CI 0.22–0.95, *I*^2^ = 89%; Figure 3). However, this effect was not seen when the analysis was restricted to the randomized controlled trials (OR 0.36, 95% CI 0.10–1.34). Sasidharan et al. (27) reported a decrease in hospital mortality but did not publish the associated data.

### Growth

Weight gain during hospitalization was measured in ten studies (11, 13, 14, 16, 17, 19, 21–25), and weight gain at follow-up post-discharge was reported in two studies (14, 29). The data was not amenable to meta-analysis due to differences in outcome reporting. Parental participation had a positive effect on weight gain in eight of the studies (80%; 11, 13, 14, 19, 21, 22, 24, 25). One study (10%) reported reduced weight gain in the intervention group compared to the control group (23), and one study (10%) found no difference between the groups (16, 17).

### Nosocomial infection

**FIGURE 4:**
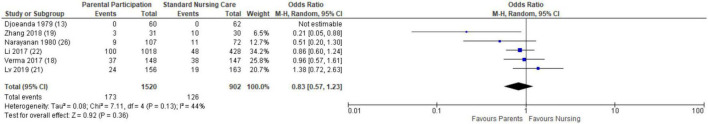
Forest plot and meta-analysis of the association between parental participation and nosocomial infection.

Eight studies measured nosocomial infection rates (13, 14, 18, 19, 21–23, 26), and six were included in the meta-analysis (13, 18, 19, 21, 22, 26). There was no difference in the odds of contracting a nosocomial infection in the intervention group compared to the control group (OR 0.83, 95% CI 0.57 – 1.23, *I*^2^ = 44%; Figure 4), and this result remained consistent when evaluating only the randomized controlled trials (OR 0.53, 95% 0.12–2.12). The two studies that were not able to be included in the meta-analysis also reported similar rates of infection between both groups (14, 23).

### Breastfeeding

**FIGURE 5:**
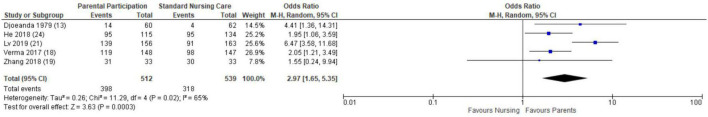
Forest plot and meta-analysis of the association between parental participation and breastfeeding during hospitalization.

Breastfeeding rates during hospitalization were reported in five studies (13, 18, 19, 21, 24). Infants whose parents participated in hospital duties were more likely to be at least partially breastfed compared to infants who received routine nursing care (OR 2.97, 95% CI 1.65 – 5.35, *I*^2^ = 65%; Figure 5). This remained true in a secondary analysis of only the randomized controlled trials (OR 2.01, 95% 1.21–3.35). An additional study evaluated breastfeeding rates three months after hospital discharge and found a higher rate of exclusive breastfeeding in the intervention group compared to the control group (29).

### Hospital readmission

**FIGURE 6:**
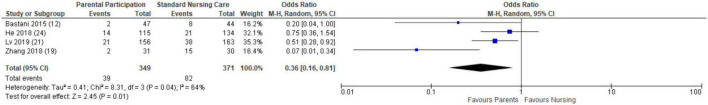
Forest plot and meta-analysis of the association between parental participation and hospital readmission.

Four studies evaluated hospital readmissions (12, 19, 21, 24). Readmissions were relatively common and ranged from 4.2 to 13.5% in intervention groups and 15.7–50.0% in control groups. Infants in the parental participation group were less likely to be readmitted to the hospital within one month post-discharge compared to neonates in the standard nursing care group (OR 0.36, 95% 0.16–0.81, *I*^2^ = 64%; Figure 6). A similar result was found when the analysis was restricted to the randomized controlled trials (OR 0.12, 95% 0.04–0.36).

### Neurodevelopment

Two studies (16, 19) assessed the infants’ neurodevelopmental outcomes. Zhang et al. (19) evaluated neurologic outcomes the day before discharge using the Neonatal Behavior Neurological Assessment (NBNA) score, while Namprom et al. (16) evaluated neurobehavioral development at day 14 and 28 of life using the Neonatal Neurobehavioral Examination (NNE). Both studies showed significant improvement in short-term neurological outcomes in the intervention group compared to the control group.

### Parental impact

Three studies evaluated family satisfaction (12, 19, 20). Meta-analysis was not performed due to differences in measurement tools. All reported enhanced satisfaction for parents who participated in the intervention compared to the control. Two studies reported on parental stress (19, 20). Zhang et al. (19) measured stress using the W.K. Zung self-assessment instrument (30), and parents who participated in their infants’ care reported less anxiety and depression compared to parents in the control group. Using the Parental Stress Scale (31), Balbino et al. (20) found that parents in the intervention group had an overall lower stress score. However, parents in the intervention group reported increased anxiety specifically related to their baby’s appearance and behavior compared to the control group. Mirlashari et al. (28) found that fathers in the intervention group reported higher levels of bonding to their infant and higher levels of self-efficacy compared to their control group. Only one study (15), assessed discharge preparedness with a validated questionnaire and found that mothers in the intervention group were more knowledgeable and confident in caring for their infant at discharge compared to mothers in the control group.

### Cost of medical care

Cost of medical care was recorded in four studies (14, 17, 21, 24), but the data was not amenable to a meta-analysis. Compared to the control group, infants in the parental participation group incurred 31% less medical expenditures in Hei et al. (14) and 43% less medical expenditures in Namprom et al. (17). The remaining two studies found no difference in cost of medical care (21, 24).

### Risk of bias

Eight studies were randomized controlled trials (11–19) and risk-of-bias was assessed using the RoB-2 tool (9) (Table 1). Ten studies were non-randomized trials (20–29) and risk of bias was assessed using the ROBINS-I tool (10) (Table 2). Eleven studies had an overall high (“high”/“serious concerns”/“critical”) risk of bias (11–13, 19–21, 24–28). The potential for confounding due to differences in prognostic factors between the intervention and the control groups was the most common reason for non-randomized trials to be assigned a high risk of bias (20, 21, 24–27). All three randomized trials that were assigned a high risk of bias had missing outcome data (11–13). Six studies had a moderate (“moderate”/“some concerns”) risk of bias (14–17, 19, 22, 23) and one study had a low risk of bias (18). One study had insufficient information to make a risk-of-bias of assessment (29).

**TABLE 1:** Risk of bias of randomized controlled trials using RoB 2 tool (9).

References	Randomization process	Deviations from the intended intervention	Missing outcome data	Measurement of the outcome	Selection of the reported result	Overall risk of bias
Arif and Arif (11)	Some concerns	Low	High	Low	Some concerns	High
Bastani et al. (12)	Some concerns	Low	High	High	Some concerns	High
Djoeanda et al. (13)	Some concerns	Low	High	High	Low	High
Hei et al. (14)	Low	Low	Some concerns	Low	Low	Some concerns
Moradi et al. (15)	Some concerns	Low	Low	Low	Low	Some concerns
Namprom et al. (16, 17)	Some concerns	Some concerns	Low	Low	Low	Some concerns
Verma et al. (18)	Low	Low	Low	Low	Low	Low
Zhang et al. (19)	Low	Low	Low	Some concerns	Low	Some concerns

**TABLE 2:** Risk of bias of non-randomized trials using ROBINS-I tool (10).

References	Confounding	Selection of participants	Classification of interventions	Deviations from intended interventions	Missing data	Measurement of outcomes	Reported result	Overall risk of bias
Balbino et al. (20)	Critical	Low	Low	Low	NI	Serious	Moderate	Critical
Bhutta et al. (23)	Moderate	Low	Low	Low	Low	Low	Moderate	Moderate
He et al. (24)	Serious	Moderate	Low	Low	Moderate	Low	Moderate	Serious
Li et al. (22)	Low	Low	Low	Low	Low	Low	Moderate	Moderate
Lv et al. (21)	Serious	Low	Low	Low	NI	Low	Moderate	Serious
Mirlashari et al. (28)	Low	Serious	Low	Low	NI	Moderate	Moderate	Serious
Mustajab et al. (25)	Serious	Low	Low	Low	NI	Low	Moderate	Serious
Narayanan et al. (29)	Low	NI	Low	NI	Low	Low	Moderate	NI
Narayanan et al. (26)	Serious	Moderate	Low	Low	Low	Low	Moderate	Serious
Sasidharan et al. (27)	Critical	Low	Low	Low	NI	Low	Moderate	Critical

## Discussion

In this meta-analysis, parental participation in neonatal hospital care in LMICs was associated with decreased mortality, increased breastfeeding and decreased hospital readmission. Additionally, included studies also suggested that parental participation had a positive impact on growth, neurodevelopmental and parental outcomes, though these outcomes could not be statistically pooled.

The importance of the parental role in neonatal hospital care is not a new concept, but the degree and range of parental participation continues to evolve. The principals of family-centered neonatal care was introduced in a seminal publication in *Pediatrics* in 1993 and focused on ensuring that parents had adequate knowledge to participate in medical decisions for their child (32). Since then, the amount of parental involvement in hospital care has grown with improved outcomes observed for both the patients and parents in high-income countries (33). A recent multi-centered, cluster-randomized trial in Canada, Australia and New Zealand demonstrated that Family Integrated Care (FICare), in which parents become a central part of the NICU care team, resulted in greater neonatal weight gain, higher rates of exclusive breastfeeding at discharge, and lower parental stress levels compared to standard NICU care (34). Adoption of this approach has not been commensurate in LMICs, despite the similar findings reported in this review. The integration of parents into the hospital care teams in LMICs may be especially valuable given very low health-worker to patient ratios which often limit the provision of high-quality nursing care.

Despite evidence regarding the benefits of parental involvement in neonatal hospital care, concerns regarding infection control continue to be used as justification for limiting parental participation (35). At the start of the COVID-19 pandemic, hospitals across the world placed restrictions on parental presence – let alone parental participation – in the NICU (36). In our review, parental participation did not increase the odds of nosocomial infection. Importantly, hand hygiene skills were specifically mentioned as being part of the training parents received in half of the studies that reported that outcome (14, 18, 19, 21, 23, 26). Bhutta et al. (23) who reported a reduction in nosocomial infection rates after patients’ mothers assumed primary nursing responsibilities suggested that having a dedicated family member providing care – rather than several medical providers touching the infant– could actually be an important factor to improve infection control. Given the lack of evidence that parental presence increases infection rates, the Global Alliance for Newborn Care, in line with WHO recommendations, has recently launched the Zero Separation campaign to promote unlimited parental access to their children hospitalized in the NICU (37) even during the COVID-19 pandemic.

One metric that was not influenced by parental participation was length-of-stay. It is likely that this is a poor surrogate for impact given the complex interaction of multiple factors that influence timing of discharge. Studies examining predictors of neonatal length-of-stay have found that intrinsic factors, such as gestational age and illness severity at admission, play a strong role in determining the date of discharge (38). Beyond intrinsic factors, organizational factors, including whether the patient census is high and whether specific services are offered in the community, sway a physician’s decision to extend or end a patient’s hospitalization (39, 40). Another possible explanation is that parental involvement results in a more optimal length-of-stay as parents become better advocates for their child’s readiness or lack of readiness for discharge. Together these possibilities may diminish any measurable impact parental involvement – however, valuable – on length of stay, thus limiting its utility as a research outcome.

Our review has several limitations that make it difficult to generalize about the impact of parental participation in LMICs. First, all of the included studies were conducted in middle-income countries and sixteen of the seventeen studies occurred in Asia. Africa, where many countries have a severely strained health workforce, neonatal mortality is high, and parental involvement may have the greatest impact, had no data available (3, 37). The lack of trials from low-income countries may reflect the difficulties of devoting already-limited healthcare resources to research. However, low-income countries have the lowest rates of education and literacy (41), making it even more prudent to study the training and support that is required to facilitate parental participation in these regions. However, Second, the majority of the studies had a high risk of bias due to methodological issues, and therefore, these results should be interpreted with caution. However, strong alignment with studies from high-income countries is encouraging. Third, especially for the non-randomized trials, there is the possibility of a selection bias whereby parents with greater resources are more likely to have the capacity to participate in their child’s care. This possibility highlights the urgency to perform high-quality studies in low-income countries where a larger percentage of families are under-resourced to determine the effects of and barriers to parental participation. Finally, substantial statistical heterogeneity in four of the five pooled analyses raises potential concerns, though this was expected given the differences in study context, design, intervention, and population. The high level of heterogeneity prevented an evaluation of the overall effect size of parental participation on neonatal and parental outcomes.

In conclusion, the global momentum for parental presence and participation during neonatal hospitalization is accelerating. Our review of parental participation in LMICs suggests promising effects on a wide range of neonatal and parental outcomes. However, the lack of data from low income countries highlights that further, high-quality research is needed to understand the barriers to parental participation in these regions and how to address them.

## Data availability statement

The original contributions presented in the study are included in the article/Supplementary material, further inquiries can be directed to the corresponding author.

## Author contributions

AR drafted the protocol, performed the data search, extraction, and analysis and drafted the manuscript. JDM drafted the protocol and performed the data search and extraction. NK-M, AT, and OK participated in the analysis and edited the manuscript. MW provided guidance on the project direction, participated in the analysis and edited the manuscript. JD conceptualized the project, approved the protocol, acted as the third senior reviewer, supervised data analysis and edited the manuscript. All authors contributed to the article and approved the submitted version.
