# Supplementary material for: Parental participation in the care of hospitalized neonates in low- and middle-income countries: A systematic review and meta-analysis
Source: Front Pediatr. 2022 Aug 25;10:987228. doi: 10.3389/fped.2022.987228 (PMC9453204; doi:10.3389/fped.2022.987228)
Supplement: Supplementary file 1 [file Data_Sheet_1.pdf]

Supplementary Material: Search Strategy

Search strategy for Medline

1. exp Infant, Newborn/ or Intensive Care Units, Neonatal/

2. (newborn* or new born or new borns or newly born or baby* or babies or premature or prematurity or preterm or pre term or low birth weight or low birthweight or VLBW or LBW or infant or infants or 'infant s' or infant's or infantile or infancy or neonat*).ti,ab.

3. (family centered care or family integrated care).ti,ab,kw.

4. ((parent* or mother* or father* or family or families or familial or maternal or paternal or caregiver* or guardian*) adj2 (involve* or empower* or program* or participat* or engage* or educat* or role* or presence or present)).ti,ab,kw.

5. Hospitals/ or Intensive Care Units, Neonatal/ or Neonatal Nursing/

6. (neonatal intensive care unit or NICU? or stepdown unit? or step down unit? or inpatient? or in-patient? or discharg* or readmission? or admission? or nursery or nurseries or nursing or ward?).ti,ab,kw.

7. (afghanistan or albania or algeria or american samoa or angola or "antigua and barbuda" or antigua or barbuda or argentina or armenia or armenian or aruba or azerbaijan or bahrain or bangladesh or barbados or republic of belarus or belarus or byelarus or belorussia or byelorussian or belize or british honduras or benin or dahomey or bhutan or bolivia or "bosnia and herzegovina" or bosnia or herzegovina or botswana or bechuanaland or brazil or brasil or bulgaria or burkina faso or burkina fasso or upper volta or burundi or urundi or cabo verde or cape verde or cambodia or kampuchea or khmer republic or cameroon or cameron or cameroun or central african republic or ubangi shari or chad or chile or china or colombia or comoros or comoro islands or iles comores or mayotte or democratic republic of the congo or democratic republic congo or congo or zaire or costa rica or "cote d’ivoire" or "cote d’ ivoire" or cote divoire or cote d ivoire or ivory coast or croatia or cuba or cyprus or czech republic or czechoslovakia or djibouti or french somaliland or dominica or dominican republic or ecuador or egypt or united arab republic or el salvador or equatorial guinea or spanish guinea or eritrea or estonia or eswatini or swaziland or ethiopia or fiji or gabon or gabonese republic or gambia or "georgia (republic)" or georgian or ghana or gold coast or gibraltar or greece or grenada or guam or guatemala or guinea or guinea bissau or guyana or british guiana or haiti or hispaniola or honduras or hungary or india or indonesia or timor or iran or iraq or isle of man or jamaica or jordan or kazakhstan or kazakh or kenya or "democratic people’s republic of korea" or republic of korea or north korea or south korea or korea or kosovo or kyrgyzstan or kirghizia or kirgizstan or kyrgyz republic or kirghiz or laos or lao pdr or "lao people's democratic republic" or latvia or lebanon or lebanese republic or lesotho or basutoland or liberia or libya or libyan arab jamahiriya or lithuania or macau or macao or "macedonia (republic)" or macedonia or madagascar or malagasy republic or malawi or nyasaland or malaysia or malay federation or malaya federation or maldives or indian ocean islands or indian ocean or mali or malta or micronesia or federated states of micronesia or kiribati or marshall islands or nauru or northern mariana islands or palau

or tuvalu or mauritania or mauritius or mexico or moldova or moldovian or mongolia or montenegro or morocco or ifni or mozambique or portuguese east africa or myanmar or burma or namibia or nepal or netherlands antilles or nicaragua or niger or nigeria or oman or muscat or pakistan or panama or papua new guinea or new guinea or paraguay or peru or philippines or philipines or phillipines or phillippines or poland or "polish people's republic" or portugal or portuguese republic or puerto rico or romania or russia or russian federation or ussr or soviet union or union of soviet socialist republics or rwanda or ruanda or samoa or pacific islands or polynesia or samoan islands or navigator island or navigator islands or "sao tome and principe" or saudi arabia or senegal or serbia or seychelles or sierra leone or slovakia or slovak republic or slovenia or melanesia or solomon island or solomon islands or norfolk island or norfolk islands or somalia or south africa or south sudan or sri lanka or ceylon or "saint kitts and nevis" or "st. kitts and nevis" or saint lucia or "st. lucia" or "saint vincent and the grenadines" or saint vincent or "st. vincent" or grenadines or sudan or suriname or surinam or dutch guiana or netherlands guiana or syria or syrian arab republic or tajikistan or tadjikistan or tadzhikistan or tadzhik or tanzania or tanganyika or thailand or siam or timor leste or east timor or togo or togolese republic or tonga or "trinidad and tobago" or trinidad or tobago or tunisia or turkey or "turkey (republic)" or turkmenistan or turkmen or uganda or ukraine or uruguay or uzbekistan or uzbek or vanuatu or new hebrides or venezuela or vietnam or viet nam or middle east or west bank or gaza or palestine or yemen or yugoslavia or zambia or zimbabwe or northern rhodesia or global south or africa south of the sahara or sub-saharan africa or subsaharan africa or africa, central or central africa or africa, northern or north africa or northern africa or magreb or maghrib or sahara or africa, southern or southern africa or africa, eastern or east africa or eastern africa or africa, western or west africa or western africa or west indies or indian ocean islands or caribbean or central america or latin america or "south and central america" or south america or asia, central or central asia or asia, northern or north asia or northern asia or asia, southeastern or southeastern asia or south eastern asia or southeast asia or south east asia or asia, western or westen asia or europe, eastern or east europe or eastern europe or developing country or developing countries or developing nation? or developing population? or developing world or less developed countr* or less developed nation? or less developed population? or less developed world or lesser developed countr* or lesser developed nation? or lesser developed population? or lesser developed world or under developed countr* or under developed nation? or under developed population? or under developed world or underdeveloped countr* or underdeveloped nation? or underdeveloped population? or underdeveloped world or middle income countr* or middle income nation? or middle income population? or low income countr* or low income nation? or low income population? or lower income countr* or lower income nation? or lower income population? or underserved countr* or underserved nation? or underserved population? or underserved world or under served countr* or under served nation? or under served population? or under served world or deprived countr* or deprived nation? or deprived population? or deprived world or poor countr* or poor nation? or poor population? or poor world or poorer countr* or poorer nation? or poorer population? or poorer world or developing economy* or less developed economy* or lesser developed economy* or under developed economy* or underdeveloped economy* or middle income economy* or low income economy* or lower income economy* or low gdp or low gnp or low gross domestic or low gross national or lower gdp or lower gnp or lower gross

domestic or lower gross national or lmic or lmics or third world or lami countr* or transitional countr* or emerging economies or emerging nation?).ti,ab,sh,kf,jn.

8. (afghan or afghans or afghani or albanian? algerian? or american samoan? or angolan? or antiguan? or barbudan? or argentine? or argentinian? or argentinean? or armenian? or aruban? or azerbaijani? or bahraini? or bangladeshi? or bangalees or bajan? or belarusian? or byelorussian? or belizean? or beninese? or bhutanese or bolivian? or bosnian? or botswana or batswana or brazilian? or brasilian? or bulgarian? or burkinabe or burkinese or burundian? or cape verdean? or cabo verdean? or cambodian? or khmer or cameroonian? or central african? or chadian? or chilean? or chinese or colombian? or comorian? or congolese or costa rican? or ivorian? or croatian? or cuban? or cypriot? or czech? or djiboutian? or dominican? or ecuadorian? or egyptian? or salvadoran? or equatorial guinean? or equatoguinean? or eritrean? or estonian? or swazi? or swati? or ethiopian? or fijian or gabonese or gabonaise or gambian? or georgian? or ghanaian? or gibraltarian? or greek? or grenadian? or guamanian? or guatemalan? or guinean? or bissau guinean? or guyanese or haitian? or honduran? or hungarian? or indian? or indonesian? or iranian? or iraqian? or iraqi? or manx or jamaican? or jordanian? or kazakhstani? or kenyan? or kirabati or kirabatian? or north korean? or korean? or kosovar? or kosovan? or kyrgyz* or lao or laotian? or latvian? or lebanese or lesothan? or lesothonian? or mosotho or basotho or liberian? or libyan? or lithuanian? or macanese or macedonian? or malagasy or madagascan? or malawian? or malaysian? or maldivian? or malian? or maltese or marshallese? or mauritanian? or mauritian? or mexican? or micronesian? or moldovan? or mongolian? or mongol or montenegrin? or moroccan? or mozambican? or burmese or myanma or namibian? or nauruan? or nepali or nepalese or netherlands antillean? or nicaraguan? or nigerien? or nigerian? or northern mariana islander? or mariana? or omani? or pakistani? or palauan? or panamanian? or papua new guinean? or paraguayan? or peruvian? or philippine? or philipine? or phillipine? or phillippine? or filipino? or filipina? or polish or pole or poles or portuguese or puerto rican? or romanian? or russian? or soviet people or soviet population or rwandan? or rwandese or ruandan? or ruandese or samoan? or sao tomean? or santomean? or saudi arabian? or saudi? or senegalese or serbian? or montenegrin? or seychellois or seychelloise? or sierra leonean? or slovak? or slovene? or solomon islander? or somali? or south african? or south sudanese or sri lankan? or ceylonese or kittitian? or nevisian? or saint lucian? or vincentian? or sudanese or surinamese? or syrian? or tajik? or tajikistani? or tanzanian? or tanganyikan? or thai or timorese? or togolese or tongan? or trinidadian? or tobagonian? or tunisian? or turk? or turkish or turkmen? or tuvaluan? or ugandan? or ukrainian? or uruguayan? or uzbek? or vanuatu* or venezuelan? or vietnamese or yemeni? or yemenite? or yemenese or yugoslav? or yugoslavian? or zambian? or zimbabwean?).ti,ab,sh,kf,jn.

9. or/1-2 [**infant]

10. or/3-4 [**intervention]

11. or/5-6 [**setting]

12. or/7-8 [**LMIC]

13. 9 and 10 and 11 and 12

Search strategy for CINAHL

1. (MH "Infant, Premature") OR (MH "Infant, Very Low Birth Weight") OR (MH "Infant, High Risk") OR (MH "Infant, Hospitalized")
2. (infant or infants or infant’s or infantile or infancy or newborn* or "new born" or "new borns" or "newly born" or neonat* or baby* or babies or premature or prematures or prematurity or preterm or preterms or "pre term" or premies or "low birth weight" or "low birthweight" or VLBW or LBW)
3. (MH "Family Centered Care") OR (MH "FCC") OR (MH "Family Integrated Care") OR (MH "FIC")
4. (family centered care or family integrated care or fcc or fic)
5. ((parent or mother or father or family or families or familial or maternal or paternal or caregiver or guardian*) N2 (involv* or empower* or program* or participat* or engage* or educat* or role* or presence or present or lead*))
6. (MH “Hospitals”) OR (MH “ Intensive Care Units”) OR (MH “Neonatal”) OR (MH “Neonatal Nursing”)
7. (neonatal intensive care unit or NICU or stepdown unit or step down unit or inpatient or in-patient or discharg* or readmission or admission or nursery or nurseries or nursing or ward)
8. (afghanistan or albania or algeria or american samoa or angola or "antigua and barbuda" or antigua or barbuda or argentina or armenia or armenian or aruba or azerbaijan or bahrain or bangladesh or barbados or republic of belarus or belarus or byelarus or belorussia or byelorussian or belize or british honduras or benin or dahomey or bhutan or bolivia or "bosnia and herzegovina" or bosnia or herzegovina or botswana or bechuanaland or brazil or brasil or bulgaria or burkina faso or burkina fasso or upper volta or burundi or urundi or cabo verde or cape verde or cambodia or kampuchea or khmer republic or cameroon or cameron or cameroun or central african republic or ubangi shari or chad or chile or china or colombia or comoros or comoro islands or iles comores or mayotte or democratic republic of the congo or democratic republic congo or congo or zaire or costa rica or "cote d’ivoire" or "cote d’ ivoire" or cote divoire or cote d ivoire or ivory coast or croatia or cuba or cyprus or czech republic or czechoslovakia or djibouti or french somaliland or dominica or dominican republic or ecuador or egypt or united arab republic or el salvador or equatorial guinea or spanish guinea or eritrea or estonia or eswatini or swaziland or ethiopia or fiji or gabon or gabonese republic or gambia or "georgia (republic)" or georgian or ghana or gold coast or gibraltar or greece or grenada or guam or guatemala or guinea or guinea bissau or guyana or british guiana or haiti or hispaniola or honduras or hungary or india or indonesia or timor or iran or iraq or isle of man or jamaica or jordan or kazakhstan or kazakh or kenya or "democratic people’s republic of korea" or republic of korea or north korea or south korea or korea or kosovo or kyrgyzstan or kirghizia or kirgizstan or kyrgyz republic or kirghiz or laos or lao pdr or "lao people's democratic republic" or latvia or lebanon or lebanese republic or lesotho or basutoland or liberia or libya or libyan arab jamahiriya or lithuania or macau or macao or "macedonia (republic)" or macedonia or madagascar or malagasy republic or malawi or nyasaland or malaysia or malay federation or malaya federation or maldives or

indian ocean islands or indian ocean or mali or malta or micronesia or federated states of micronesia or kiribati or marshall islands or nauru or northern mariana islands or palau or tuvalu or mauritania or mauritius or mexico or moldova or moldovian or mongolia or montenegro or morocco or ifni or mozambique or portuguese east africa or myanmar or burma or namibia or nepal or netherlands antilles or nicaragua or niger or nigeria or oman or muscat or pakistan or panama or papua new guinea or new guinea or paraguay or peru or philippines or philipines or phillipines or phillippines or poland or "polish people's republic" or portugal or portuguese republic or puerto rico or romania or russia or russian federation or ussr or soviet union or union of soviet socialist republics or rwanda or ruanda or samoa or pacific islands or polynesia or samoan islands or navigator island or navigator islands or "sao tome and principe" or saudi arabia or senegal or serbia or seychelles or sierra leone or slovakia or slovak republic or slovenia or melanesia or solomon island or solomon islands or norfolk island or norfolk islands or somalia or south africa or south sudan or sri lanka or ceylon or "saint kitts and nevis" or "st. kitts and nevis" or saint lucia or "st. lucia" or "saint vincent and the grenadines" or saint vincent or "st. vincent" or grenadines or sudan or suriname or surinam or dutch guiana or netherlands guiana or syria or syrian arab republic or tajikistan or tadjikistan or tadzhikistan or tadzhik or tanzania or tanganyika or thailand or siam or timor leste or east timor or togo or togolese republic or tonga or "trinidad and tobago" or trinidad or tobago or tunisia or turkey or "turkey (republic)" or turkmenistan or turkmen or uganda or ukraine or uruguay or uzbekistan or uzbek or vanuatu or new hebrides or venezuela or vietnam or viet nam or middle east or west bank or gaza or palestine or yemen or yugoslavia or zambia or zimbabwe or northern rhodesia or global south or africa south of the sahara or sub-saharan africa or subsaharan africa or africa, central or central africa or africa, northern or north africa or northern africa or magreb or maghrib or sahara or africa, southern or southern africa or africa, eastern or east africa or eastern africa or africa, western or west africa or western africa or west indies or indian ocean islands or caribbean or central america or latin america or "south and central america" or south america or asia, central or central asia or asia, northern or north asia or northern asia or asia, southeastern or southeastern asia or south eastern asia or southeast asia or south east asia or asia, western or westen asia or europe, eastern or east europe or eastern europe or developing country or developing countries or developing nation or developing population or developing world or less developed countr* or less developed nation or less developed population or less developed world or lesser developed countr* or lesser developed nation or lesser developed population or lesser developed world or under developed countr* or under developed nation or under developed population or under developed world or underdeveloped countr* or underdeveloped nation or underdeveloped population or underdeveloped world or middle income countr* or middle income nation or middle income population or low income countr* or low income nation or low income population or lower income countr* or lower income nation or lower income population or underserved countr* or underserved nation or underserved population or underserved world or under served countr* or under served nation or under served population or under served world or deprived countr* or deprived nation or deprived population or deprived

world or poor countr* or poor nation or poor population or poor world or poorer countr* or poorer nation or poorer population or poorer world or developing economy* or less developed economy* or lesser developed economy* or under developed economy* or underdeveloped economy* or middle income economy* or low income economy* or lower income economy* or low gdp or low gnp or low gross domestic or low gross national or lower gdp or lower gnp or lower gross domestic or lower gross national or lmic or lmics or third world or lami countr* or transitional countr* or emerging economies or emerging nation) (LMIC)

9. (afghan or afghans or afghani or albanian algerian or american samoan or angolan or antiguan or barbudan or argentine or argentinian or argentinean or armenian or aruban or azerbaijani or bahraini or bangladeshi or bangalees or bajan or belarusian or byelorussian or belizean or beninese or bhutanese or bolivian or bosnian or botswana or batswana or brazilian or brasilian or bulgarian or burkinabe or burkinese or burundian or cape verdean or cabo verdean or cambodian or khmer or cameroonian or central african or chadian or chilean or chinese or colombian or comorian or congolese or costa rican or ivorian or croatian or cuban or cypriot or czech or djiboutian or dominican or ecuadorian or egyptian or salvadoran or equatorial guinean or equatoguinean or eritrean or estonian or swazi or swati or ethiopian or fijian or gabonese or gabonaise or gambian or georgian or ghanaian or gibraltarian or greek or grenadian or guamanian or guatemalan or guinean or bissau guinean or guyanese or haitian or honduran or hungarian or indian or indonesian or iranian or iraqian or iraqi or manx or jamaican or jordanian or kazakhstani or kenyan or kirabati or kirabatian or north korean or korean or kosovar or kosovan or kyrgyz* or lao or laotian or latvian or lebanese or lesothan or lesothonian or mosotho or basotho or liberian or libyan or lithuanian or macanese or macedonian or malagasy or madagascan or malawian or malaysian or maldivian or malian or maltese or marshallese or mauritanian or mauritian or mexican or micronesian or moldovan or mongolian or mongol or montenegrin or moroccan or mozambican or burmese or myanma or namibian or nauruan or nepali or nepalese or netherlands antillean or nicaraguan or nigerien or nigerian or northern mariana islander or mariana or omani or pakistani or palauan or panamanian or papua new guinean or paraguayan or peruvian or philippine or philipine or phillipine or phillippine or filipino or filipina or polish or pole or poles or portuguese or puerto rican or romanian or russian or soviet people or soviet population or rwandan or rwandese or ruandan or ruandese or samoan or sao tomean or santomean or saudi arabian or saudi or senegalese or serbian or montenegrin or seychellois or seychelloise or sierra leonean or slovak or slovene or solomon islander or somali or south african or south sudanese or sri lankan or ceylonese or kittitian or nevisian or saint lucian or vincentian or sudanese or surinamese or syrian or tajik or tajikistani or tanzanian or tanganyikan or thai or timorese or togolese or tongan or trinidadian or tobagonian or tunisian or turk or turkish or turkmen or tuvaluan or ugandan or ukrainian or uruguayan or uzbek or vanuatu* or venezuelan or vietnamese or yemeni or yemenite or yemenese or yugoslav or yugoslavian or zambian or zimbabwean) (LMIC)
10. (S1 or S2) AND (S3 or S4 or S5) AND (S6 or S7) AND (S8 or S9)

Search Strategy for CENTRAL

1. [mh “Infant, Newborn”]
2. [mh ^”Intensive Care Units, Neonatal”]
3. (newborn* or new born or new borns or newly born or baby* or babies or premature or prematurity or preterm or pre term or low birth weight or low birthweight or VLBW or LBW or infant or infants or 'infant s' or infant's or infantile or infancy or neonat*):ti,ab,kw.
4. (family centered care or family integrated care):ti,ab,kw.
5. ((parent* or mother* or father* or family or families or familial or maternal or paternal or caregiver* or guardian*) NEAR/2 (involve* or empower* or program* or participat* or engage* or educat* or role* or presence or present)):ti,ab,kw
6. [mh “Hospitals”]
7. [mh ^Intensive Care Units, Neonatal”]
8. [mh ^”Neonatal Nursing”]
9. (neonatal intensive care unit or NICU? or stepdown unit? or step down unit? or inpatient? or in-patient? or discharg* or readmission? or admission? or nursery or nurseries or nursing or ward?):ti,ab,kw
10. ((afghanistan OR albania OR algeria OR "american samoa" OR angola OR "antigua and barbuda" OR antigua OR barbuda OR argentina OR armenia OR armenian OR aruba OR azerbaijan OR bahrain OR bangladesh ORbarbados OR "republic of belarus" OR belarus OR byelarus OR belorussia OR byelorussian OR belize OR "british honduras" OR benin OR dahomey OR bhutan OR bolivia OR "bosnia and herzegovina" OR bosnia OR herzegovina OR botswana OR bechuanaland OR brazil OR brasil OR bulgaria OR "burkina faso" OR "burkina fasso" OR "upper volta" OR burundi OR urundi OR "cabo verde" OR "cape verde" OR cambodia OR kampuchea OR "khmer republic" OR cameroon OR cameron OR cameroun OR "central african republic" OR "ubangi shari" OR chad OR chile OR china OR colombia OR comoros OR "comoro islands" OR "iles comores" OR mayotte OR "democratic republic of the congo" OR "democratic republic congo" OR congo OR zaire OR "costa rica" OR "cote d’ivoire" OR "cote d’ ivoire" OR "cote divoire" OR "cote d ivoire" OR "ivory coast" OR croatia OR cuba OR cyprus OR "czech republic" OR czechoslovakia OR djibouti OR "french somaliland" OR dominica OR "dominican republic" OR ecuador OR egypt OR "united arab republic" OR "el salvador" OR "equatorial guinea"OR "spanish guinea" OR eritrea OR estonia OR eswatini OR swaziland OR ethiopia OR fiji OR gabon OR "gabonese republic" OR gambia OR "georgia (republic)" OR georgia OR georgian OR ghana OR "gold coast" OR gibraltar OR greece OR grenada OR guam OR guatemala OR guinea OR "guinea bissau" OR guyana OR "british guiana" OR haiti OR hispaniola OR honduras OR hungary OR india OR indonesia OR timor OR iran OR iraq OR "isle of man" OR jamaica OR jordan OR kazakhstan OR kazakh OR kenya OR "democratic people’s republic of korea" OR "republic of korea" OR north korea OR south korea OR korea OR kosovo OR kyrgyzstanOR kirghizia OR kirgizstan OR "kyrgyz republic" OR kirghiz OR laos OR "lao pdr" OR "lao people's democratic republic" OR latvia OR lebanon OR "lebanese republic" OR lesotho OR basutoland OR liberia OR libya OR "libyan arab jamahiriya" OR lithuania OR macau OR macao OR "macedonia (republic)" OR macedonia OR

madagascarOR "malagasy republic" OR malawi OR nyasaland OR malaysia OR "malay federation" OR "malaya federation" OR maldives OR "indian ocean islands" OR "indian ocean" OR mali OR malta OR micronesia OR "federated states of micronesia" OR kiribati OR "marshall islands" OR nauru OR "northern mariana islands" OR palau OR tuvalu OR mauritania OR mauritius OR mexico OR moldova OR moldovian OR mongolia OR montenegro OR moroccoOR ifni OR mozambique OR "portuguese east africa" OR myanmar OR burma OR namibia OR nepal OR "netherlands antilles" OR nicaragua OR niger OR nigeria OR oman OR muscat OR pakistan OR panama OR "papua new guinea" OR paraguay OR peru OR philippines OR philipines OR phillipines OR phillippines OR poland OR "polish people's republic" OR portugal OR "portuguese republic" OR "puerto rico" OR romania OR russia OR "russian federation" OR ussr OR "soviet union" OR "union of soviet socialist republics" OR rwanda OR ruanda OR samoa OR "pacific islands" OR polynesia OR "samoan islands" OR "navigator island" OR "navigator islands" OR "sao tome and principe" OR "saudi arabia" OR senegal OR serbia OR seychelles OR "sierra leone" OR slovakia OR "slovak republic" OR slovenia OR melanesia OR "solomon island" OR "solomon islands" OR "norfolk island" OR "norfolk islands" OR somalia OR "south africa" OR "south sudan" OR "sri lanka" OR ceylon OR "saint kitts and nevis" OR "st. kitts and nevis" OR "saint lucia" OR "st. lucia" OR "saint vincent and the grenadines" OR "saint vincent" OR "st. vincent" OR grenadines OR sudan OR suriname OR surinam OR "dutch guiana" OR "netherlands guiana" OR syria OR "syrian arab republic" OR tajikistan OR tadjikistan OR tadzhikistan OR tadzhik OR tanzania OR tanganyika OR thailand OR siam OR "timor leste" OR "east timor" OR togo OR "togolese republic" OR tonga OR "trinidad and tobago" OR trinidad OR tobago OR tunisia OR turkey OR "turkey (republic)" OR turkmenistan OR turkmen OR uganda OR ukraine OR uruguay OR uzbekistan OR uzbek OR vanuatu OR "new hebrides" OR venezuela OR vietnam OR "viet nam" OR "middle east" OR "west bank" OR gaza OR palestine OR yemen OR yugoslavia OR zambia OR zimbabwe OR "northern rhodesia" OR "global south" OR "africa south of the sahara" OR "sub saharan africa" OR "subsaharan africa" OR "africa, central" OR "central africa" OR "africa, northern OR "north africa" OR "northern africa" OR magreb OR maghrib OR sahara-OR "africa, southern OR "southern africa" OR "africa, eastern OR "east africa" OR "eastern africa" OR "africa, western OR west africa OR western africa OR "west indies" OR "indian ocean islands" OR caribbean OR "central america"OR "latin america" OR "south and central america" OR "south america" OR "asia, central" OR "central asia" OR "asia, northern" OR "north asia" OR "northern asia" OR "asia, southeastern" OR "southeastern asia" OR "south eastern asia" OR "southeast asia" OR "south east asia" OR "asia, western" OR "westen asia" OR "europe, eastern" OR "east europe" OR "eastern europe" OR "developing country" OR "developing countries" OR "developing nation" OR "developing nations" OR "developing population" OR "developing populations" OR "developing world" OR "less developed country" OR "less developed countries" OR "less developed nation" OR "less developed nations" OR "less developed population" OR "less developed populations" OR "less developed world" OR "lesser developed country" OR "lesser developed countries" OR "lesser developed nation" OR "lesser developed nations"

OR "lesser developed population" OR "lesser developed populations" OR "lesser developed world" OR "under developed country" OR "under developed countries" OR "under developed nation" OR "under developed nations" OR "under developed population" OR "under developed populations" OR "under developed world" OR "underdeveloped country" OR "underdeveloped countries" OR "underdeveloped nation" OR "underdeveloped nations" OR "underdeveloped population" OR "underdeveloped populations" OR "underdeveloped world" OR "middle income country" OR "middle income countries" OR "middle income nation" OR "middle income nations" OR "middle income population" OR "middle income populations" OR "low income country" OR "low income countries" OR "low income nation" OR "low income nations" OR "low income population" OR "low income populations" OR "lower income country" OR "lower income countries" OR "lower income nation" OR "lower income nations" OR "lower income population" OR "lower income populations" OR "underserved country" OR "underserved countries" OR "underserved nation" OR "underserved nations" OR "underserved population" OR "underserved populations" OR "underserved world" OR "under served country" OR "under served countries" OR "under served nation" OR "under served nations" OR "under served population" OR "under served populations" OR "under served world" OR "deprived country" OR "deprived countries" OR "deprived nation" OR "deprived nations" OR "deprived population" OR "deprived populations" OR "deprived world" OR "poor country" OR "poor countries" OR "poor nation" OR "poor nations" OR "poor population" OR "poor populations" OR "poor world" OR "poorer country" OR "poorer countries" OR "poorer nation" OR "poorer nations" OR "poorer population" OR "poorer populations" OR "poorer world" OR "developing economy" OR "developing economies" OR "less developed economy" OR "less developed economies" OR "lesser developed economy" OR "lesser developed economies" OR "under developed economy" OR "under developed economies" OR "underdeveloped economy" OR "underdeveloped economies" OR "middle income economy" OR "middle income economies" OR "low income economy" OR "low income economies" OR "lower income economy" OR "lower income economies" OR "low gdp" OR "low gnp" OR "low gross domestic" OR "low gross national" OR "lower gdp" OR "lower gnp" OR "lower gross domestic" OR "lower gross national" OR lmic OR lmics OR "third world" OR "lami country" OR "lami countries" OR "transitional country" OR "transitional countries" OR "emerging economies" OR "emerging nation" OR "emerging nations"):ti,ab,kw

11. (afghan OR afghans OR afghani OR albanian OR albanians OR algerian OR algerians OR "american samoan" OR "american samoans" OR angolan OR angolans OR antiguan OR antiguans OR barbudan OR berbudans OR argentine OR argentines OR argentinian OR argentinians OR argentinean OR argentineans OR armenian OR armenians OR aruban OR arubans OR azerbaijani OR azerbaijanis OR bahraini OR bahrainis OR bangladeshi OR bangladeshis OR bangalees OR bajan OR bajans OR belarusian OR belarusians OR byelorussian OR byelorussians OR belizean OR belizeans OR beninese OR benineses OR bhutanese OR bolivian OR bolivians OR bosnian OR bosnians OR botswana OR batswana OR brazilian OR brazilians OR brasilian OR brasilians OR bulgarian OR bulgarians OR burkinabe OR burkinese OR burundian OR burundians OR

"cape verdean" OR "cape verdeans" OR "cabo verdean" OR "cabo verdeans" OR cambodian OR cambodians OR khmer OR cameroonian OR cameroonians OR "central african" OR "central africans" OR chadian OR chadians OR chilean OR chileans OR chinese OR colombian OR colombians OR comorian OR comorians OR congolese OR "costa rican" OR "costa ricans" OR ivorian OR ivorians OR croatian OR croatians OR cuban OR cubans OR cypriot OR cypriots OR czech OR czechs OR djiboutian OR djiboutians OR dominican OR dominicans OR ecuadorian OR ecuadorians OR egyptian OR egyptians OR salvadoran OR salvadorans OR "equatorial guinean" OR "equatorial guineans" OR equatoguinean OR equatoguineans OR eritrean OR eritreans OR estonian OR estonians OR swazi OR swazis OR swati OR swatis OR ethiopian OR ethiopians OR fijian OR fijians OR gabonese OR gabonaise OR gambian OR gambians OR georgian OR georgians OR ghanaian OR ghanaians OR gibraltarian OR gibraltarians OR greek OR greeks OR grenadian OR grenadians OR guamanian OR guamanians OR guatemalan OR guatemalans OR guinean OR guineans OR "bissau guinean" OR "bissau guineans" OR guyanese OR haitian OR haitians OR honduran OR hondurans OR hungarian OR hungarians OR indian OR indians OR indonesian OR indonesians OR iranian OR iranians OR iraqian OR iraqians OR iraqi OR iraqis OR manx OR jamaican OR jamaicans OR jordanian OR jordanians OR kazakhstani OR kazakhstanis OR kenyan OR kenyans OR kirabati OR kirabatian OR kirabatians OR "north korean" OR "north koreans" OR korean OR koreans OR kosovar OR kosovars OR kosovan OR kosovans OR kyrgyzstani OR kyrgyzstanis OR kyrgyz OR lao OR laotian OR laotians OR latvian OR latvians OR lebanese OR lesothan OR lesothans OR lesothonian OR lesothonians OR mosotho OR basotho OR liberian OR liberians OR libyan OR libyans OR lithuanian OR lithuanians OR macanese OR macedonian OR macedonians OR malagasy OR madagascan OR madagascans OR malawian OR malawians OR malaysian OR malaysians OR maldivian OR maldivians OR malian OR malians OR maltese OR marshallese OR marshalleses OR mauritanian OR mauritanians OR mauritian OR mauritians OR mexican OR mexicans OR micronesian OR micronesians OR moldovan OR moldovans OR mongolian OR mongolians OR mongol OR montenegrin OR montenegrins OR moroccan OR moroccans OR mozambican OR mozambicans OR burmese OR myanma OR namibian OR namibians OR nauruan OR nauruans OR nepali OR nepalese OR "netherlands antillean" OR "netherlands antilleans" OR nicaraguan OR nicaraguans OR nigerien OR nigeriens OR nigerian OR nigerians OR "northern mariana islander" OR "northern mariana islanders" OR mariana OR marianas OR omani OR omanis OR pakistani OR pakistanis OR palauan OR palauans OR panamanian OR panamanians OR "papua new guinean" OR "papua new guineans" OR paraguayan OR paraguayans OR peruvian OR peruvians OR philippine OR philippines OR philipine OR philipines OR phillipine OR phillipines OR phillippine OR phillippines OR filipino OR filipinos OR filipina OR filipinas OR polish OR pole OR poles OR portuguese OR "puerto rican" OR "puerto ricans" OR romanian OR romanians OR russian OR russians OR "soviet people" OR "soviet population" OR rwandan OR rwandans OR rwandese OR ruandan OR ruandans OR ruandese OR samoan OR samoans OR "sao tomean" OR "sao tomeans" OR santomean OR santomeans OR "saudi arabian" OR "saudi arabians" OR

saudi OR saudis OR senegalese OR serbian OR serbians OR montenegrin OR montenegrins OR seychellois OR seychelloise OR seychelloises OR "sierra leonean" OR "sierra leoneans" OR slovak OR slovaks OR slovene OR slovenes OR "solomon islander" OR "solomon islanders" OR somali OR somalis OR "south african" OR "south africans" OR "south sudanese" OR "sri lankan" OR "sri lankans" OR ceylonese OR kittitian OR kittitians OR nevisian OR nevisians OR "saint lucian" OR "saint lucians" OR vincentian OR vincentians OR sudanese OR surinamese OR surinameses OR syrian OR syrians OR tajik OR tajiks OR tajikistani OR tajikistanis OR tanzanian OR tanzanians OR tanganyikan OR tanganyikans OR thai OR timorese OR timoreses OR togolese OR tongan OR tongans OR trinidadian OR trinidadians OR tobagonian OR tobagonians OR tunisian OR tunisians OR turk OR turks OR turkish OR turkmen OR turkmens OR tuvaluan OR tuvaluans OR ugandan OR ugandans OR ukrainian OR ukrainians uruguayan OR uruguayans OR uzbek OR uzbeks OR vanuatu OR vanuatuan OR vanuatuans OR venezuelan OR venezuelans OR vietnamese OR yemeni OR yemenis OR yemenite OR yemenites OR yemenese OR yugoslav OR yugoslavs OR yugoslavian OR yugoslavians OR zambian OR zambians OR zimbabwean OR zimbabweans)**:ti,ab,kw**

12. #1 OR #2 OR #3
13. #4 OR #5
14. #6 OR #7 OR #9
15. #9 OR #10
16. 12 AND 13 AND 14 AND 15

Search strategy for EMBASE

1. infant/ or baby/ or high risk infant/ or hospitalized infant/ or newborn/
2. (newborn* or new born or new borns or newly born or baby* or babies or premature or prematurity or preterm or pre term or low birth weight or low birthweight or VLBW or LBW or infant or infants or 'infant s' or infant's or infantile or infancy or neonat*).tw,kw.
3. family centered care/
4. (family centered care or family integrated care or FCC or FIC).tw,kw.
5. ((parent* or mother* or father* or family or families or familial or maternal or paternal or caregiver* or guardian*) adj2 (involve* or empower* or program* or participat* or engage* or educat* or role* or presence or present or lead or lead*)).tw,kw.
6. exp Hospitals/
7. exp neonatal intensive care unit/
8. newborn intensive care nursing/
9. 6. (neonatal intensive care unit or NICU? or stepdown unit? or step down unit? or inpatient? or in-patient? or discharg* or readmission? or admission? or nursery or nurseries or nursing or ward?).tw,kw.
10. (**afghanistan** OR **albania** OR **algeria** OR **american samoa** OR **angola** OR **"antigua and barbuda"** OR antigua OR barbuda OR **argentina** OR **armenia** OR armenian OR **aruba** OR **azerbaijan** OR **bahrain** OR **bangladesh** OR **barbados** OR republic of belarus OR **belarus** OR byelarus OR belorussia OR byelorussian OR **belize** OR british honduras OR **benin** OR dahomey OR **bhutan** OR **bolivia** OR **"bosnia and**

herzegovina" OR bosnia OR herzegovina OR **botswana** OR bechuanaland OR **brazil** OR brasil OR **bulgaria** OR **burkina faso** OR burkina fasso OR upper volta OR **burundi** OR urundi OR cabo verde OR **cape verde** OR **cambodia** OR kampuchea OR khmer republic OR **cameroon** OR cameron OR cameroun OR **central african republic** OR ubangi shari OR **chad** OR **chile**OR **china** OR **colombia** OR **comoros** OR comoro islands OR iles comores OR mayotte OR democratic republic of the congo OR **democratic republic congo** OR **congo** OR zaire OR **costa rica** OR **"cote d'ivoire"** OR "cote d' ivoire" OR cote divoire OR cote d ivoire OR ivory coast OR **croatia** OR **cuba** OR **cyprus** OR **czech republic** OR czechoslovakia OR **djibouti** OR french somaliland OR **dominica** OR **dominican republic** OR **ecuador** OR **egypt**OR united arab republic OR **el salvador** OR **equatorial guinea** OR spanish guinea OR **eritrea** OR **estonia** OR eswatini OR **swaziland** OR **ethiopia** OR **fiji** OR **gabon** OR gabonese republic OR **gambia** OR **"georgia (republic)"**OR georgian OR **ghana** OR gold coast OR **gibraltar** OR **greece** OR **grenada** OR **guam** OR **guatemala** OR **guinea**OR **guinea bissau** OR **guyana** OR british guiana OR **haiti** OR hispaniola OR **honduras** OR **hungary** OR **india** OR **indonesia** OR timor OR **iran** OR **iraq** OR **isle of man** OR **jamaica** OR **jordan** OR **kazakhstan** OR kazakh OR **kenya** OR "democratic people's republic of korea" OR republic of korea OR **north korea** OR **south korea** OR korea OR **kosovo** OR **kyrgyzstan** OR kirghizia OR kirgizstan OR kyrgyz republic OR kirghiz OR **laos** OR lao pdr OR "lao people's democratic republic" OR **latvia** OR **lebanon** OR lebanese republic OR **lesotho** OR basutoland OR **liberia**OR libya OR **libyan arab jamahiriya** OR **lithuania** OR **macau** OR macao OR **"macedonia (republic)"** OR macedonia OR **madagascar** OR malagasy republic OR **malawi** OR nyasaland OR **malaysia** OR malay federation OR malaya federation OR maldives OR indian ocean islands OR **indian ocean** OR **mali** OR **malta** OR micronesia OR **federated states of micronesia** OR **kiribati** OR marshall islands OR nauru OR northern mariana islands OR palau OR tuvalu OR **mauritania** OR **mauritius** OR **mexico** OR **moldova** OR moldovian OR **mongolia** OR montenegro OR **"montenegro (republic)"** OR **morocco** OR ifni OR **mozambique** OR portuguese east africa OR**myanmar** OR burma OR **namibia** OR **nepal** OR **netherlands antilles** OR **nicaragua** OR **niger** OR **nigeria** OR **oman** OR muscat OR **pakistan** OR **panama** OR **papua new guinea** OR new guinea OR **paraguay** OR **peru** OR **philippines** OR philipines OR phillipines OR phillippines OR **poland** OR "polish people's republic" OR **portugal** OR portuguese republic OR **puerto rico** OR **romania** OR russia OR **russian federation** OR ussr OR soviet union OR union of soviet socialist republics OR **rwanda** OR ruanda OR **samoa** OR pacific islands OR polynesia OR samoan islands OR navigator island OR navigator islands OR **"sao tome and principe"** OR **saudi arabia** OR **senegal** OR **serbia** OR **seychelles** OR **sierra leone** OR **slovakia** OR slovak republic OR **slovenia** OR **melanesia** OR solomon island OR solomon islands OR norfolk island OR norfolk islands OR **somalia** OR **south africa** OR **south sudan** OR **sri lanka** OR ceylon OR **"saint kitts and nevis"** OR "st. kitts and nevis" OR **saint lucia** OR "st. lucia" OR **"saint vincent and the grenadines"** OR saint vincent OR "st. vincent" OR grenadines OR **sudan** OR **suriname** OR surinam OR dutch guiana OR netherlands guiana OR syria OR **syrian arab republic** OR **tajikistan** OR tadjikistan OR tadzhikistan

OR tadzhik OR **tanzania** OR tanganyika OR **thailand** OR siam OR **timor leste** OR east timor OR **togo** OR togolese republic OR **tonga** OR **"trinidad and tobago"** OR trinidad OR tobago OR **tunisia** OR turkey OR **"turkey (republic)"** OR **turkmenistan** OR turkmen OR **uganda** OR **ukraine** OR **uruguay** OR **uzbekistan** OR uzbek OR **vanuatu** OR new hebrides OR **venezuela** OR vietnam OR **viet nam** OR middle east OR west bank OR gaza OR **palestine** OR **yemen** OR **yugoslavia** OR **zambia** OR **zimbabwe** OR northern rhodesia OR global south OR **africa south of the sahara** OR "sub saharan africa" OR subsaharan africa OR **africa, central** OR central africa OR **africa, northern** OR north africa OR northern africa OR magreb OR maghrib OR sahara OR **africa, southern** OR southern africa OR **africa, eastern** OR east africa OR eastern africa OR **africa, western** OR west africa OR western africa OR **west indies** OR **indian ocean islands** OR **caribbean region** OR caribbean islands OR caribbean OR **central america** OR latin america OR "south and central america" OR **south america** OR **asia, central** OR central asia OR **asia, northern** OR north asia OR northern asia OR **asia, southeastern** OR southeastern asia OR south eastern asia OR southeast asia OR south east asia OR **asia, western** OR westen asia OR **europe, eastern** OR east europe OR eastern europe OR **developing country** OR developing countries OR developing nation? OR developing population? OR developing world OR less developed countr* OR less developed nation? OR less developed population? OR less developed world OR lesser developed countr* OR lesser developed nation? OR lesser developed population? OR lesser developed world OR under developed countr* OR under developed nation? OR under developed population? OR under developed world OR underdeveloped countr* OR underdeveloped nation? OR underdeveloped population? OR underdeveloped world OR middle income countr* OR middle income nation? OR middle income population? OR low income countr* OR low income nation? OR low income population? OR lower income countr* OR lower income nation? OR lower income population? OR underserved countr* OR underserved nation? OR underserved population? OR underserved world OR under served countr* OR under served nation? OR under served population? OR under served world OR deprived countr* OR deprived nation? OR deprived population? OR deprived world OR poor countr* OR poor nation? OR poor population? OR poor world OR poorer countr* OR poorer nation? OR poorer population? OR poorer world OR developing economy* OR less developed economy* OR lesser developed economy* OR under developed economy* OR underdeveloped economy* OR middle income economy* OR low income economy* OR lower income economy* OR low gdp OR low gnp OR low gross domestic OR low gross national OR lower gdp ORlower gnp OR lower gross domestic OR lower gross national OR lmic OR lmics OR third world OR lami countr* OR transitional countr* OR emerging economies OR emerging nation?)**.ti,ab,sh,kw.**

11. (afghan OR afghans OR afghani OR albanian? algerian? OR american samoan? OR angolan? OR antiguan? OR barbudan? OR argentine? OR argentinian? OR argentinean? OR armenian? OR aruban? OR azerbaijani? OR bahraini? OR bangladeshi? OR bangalees OR bajan? OR belarusian? OR byelorussian? OR belizean? OR beninese? OR

bhutanese OR bolivian? OR bosnian? OR botswana OR batswana OR brazilian? OR brasilian? OR bulgarian? OR burkinabe OR burkinese OR burundian? OR cape verdean? OR cabo verdean? OR cambodian? OR khmer OR cameroonian? OR central african? OR chadian? OR chilean? OR chinese OR colombian? OR comorian? OR congolese OR costa rican? OR ivorian? OR croatian? OR cuban? OR cypriot? OR czech? OR djiboutian? OR dominican? OR ecuadorian? OR egyptian? OR salvadoran? OR equatorial guinean? OR equatoguinean? OR eritrean? OR estonian? OR swazi? OR swati? OR ethiopian? OR fijian OR gabonese OR gabonaise OR gambian? OR georgian? OR ghanaian? OR gibraltarian? OR greek? OR grenadian? OR guamanian? OR guatemalan? OR guinean? OR bissau guinean? OR guyanese OR haitian? OR honduran? OR hungarian? OR indian? OR indonesian? OR iranian? OR iraqian? OR iraqi? OR manx OR jamaican? OR jordanian? OR kazakhstani? OR kenyan? OR kirabati OR kirabatian? OR north korean? OR korean? OR kosovar? OR kosovan? OR kyrgyz* OR lao OR laotian? OR latvian? OR lebanese OR lesothan? OR lesothonian? OR mosotho OR basotho OR liberian? OR libyan? OR lithuanian? OR macanese OR macedonian? OR malagasy OR madagascan? OR malawian? OR malaysian? OR maldivian? OR malian? OR maltese OR marshallese? OR mauritanian? OR mauritian? OR mexican? OR micronesian? OR moldovan? OR mongolian? OR mongol OR montenegrin? OR moroccan? OR mozambican? OR burmese OR myanma OR namibian? OR nauruan? OR nepali OR nepalese OR netherlands antillean? OR nicaraguan? OR nigerien? OR nigerian? OR northern mariana islander? OR mariana? OR omani? OR pakistani? OR palauan? OR panamanian? OR papua new guinean? OR paraguayan? OR peruvian? OR philippine? OR philipine? OR phillipine? OR phillippine? OR filipino? OR filipina? OR polish OR pole OR poles OR portuguese OR puerto rican? OR romanian? OR russian? OR soviet people OR soviet population OR rwandan? OR rwandese OR ruandan? OR ruandese OR samoan? OR sao tomean? OR santomean? OR saudi arabian? OR saudi? OR senegalese OR serbian? OR montenegrin? OR seychellois OR seychelloise? OR sierra leonean? OR slovak? OR slovene? OR solomon islander? OR somali? OR south african? OR south sudanese OR sri lankan? OR ceylonese OR kittitian? OR nevisian? OR saint lucian? OR vincentian? OR sudanese OR surinamese? OR syrian? OR tajik? OR tajikistani? OR tanzanian? OR tanganyikan? OR thai OR timorese? OR togolese OR

tongan? OR trinidadian? OR tobagonian? OR tunisian? OR turk? OR turkish OR turkmen? OR tuvaluan? OR ugandan? OR ukrainian? OR uruguayan? OR uzbek? OR vanuatu* OR venezuelan? OR vietnamese OR yemeni? OR yemenite? OR yemenese OR yugoslav? OR yugoslavian? OR zambian? OR zimbabwean?)**.ti,ab,sh,kw.**

12. 1 or 2 [**infant]
13. 3 or 4 or 5 [**intervention]
14. 6 or 7 or 8 or 9 [**setting]
15. 10 or 11 [**LMIC]
16. 12 and 13 and 14 and 15

Search Strategy for Web of Science

1. TS=(newborn* or new born or new borns or newly born or baby* or babies or premature or prematurity or preterm or pre term or low birth weight or low birthweight or VLBW or LBW or infant or infants or 'infant s' or infant's or infantile or infancy or neonat*)
2. TS=(family centered care or family integrated care or FCC or FIC)
3. TS=((parent* or mother* or father* or family or families or familial or maternal or paternal or caregiver* or guardian*) NEAR/2 (involve* or empower* or program* or participat* or engage* or educat* or role* or presence or present))
4. TS=(neonatal intensive care unit or NICU? or stepdown unit? or step down unit? or inpatient? or in-patient? or discharg* or readmission? or admission? or nursery or nurseries or nursing or ward?)
5. TS=(afghanistan or albania or algeria or american samoa or angola or "antigua and barbuda" or antigua or barbuda or argentina or armenia or armenian or aruba or azerbaijan or bahrain or bangladesh or barbados or republic of belarus or belarus or byelarus or belorussia or byelorussian or belize or british honduras or benin or dahomey or bhutan or bolivia or "bosnia and herzegovina" or bosnia or herzegovina or botswana or bechuanaland or brazil or brasil or bulgaria or burkina faso or burkina fasso or upper volta or burundi or urundi or cabo verde or cape verde or cambodia or kampuchea or khmer republic or cameroon or cameron or cameroun or central african republic or ubangi shari or chad or chile or china or colombia or comoros or comoro islands or iles comores or mayotte or democratic republic of the congo or democratic republic congo or congo or zaire or costa rica or "cote d’ivoire" or "cote d’ ivoire" or cote divoire or cote d ivoire or ivory coast or croatia or cuba or cyprus or czech republic or czechoslovakia or djibouti or french somaliland or dominica or dominican republic or ecuador or egypt or united arab republic or el salvador or equatorial guinea or spanish guinea or eritrea or estonia or eswatini or swaziland or ethiopia or fiji or gabon or gabonese republic or gambia or "georgia (republic)" or georgian or ghana or gold coast or gibraltar or greece or grenada or guam or guatemala or guinea or guinea bissau or guyana or british guiana or haiti or hispaniola or honduras or hungary or india or indonesia or timor or iran or iraq or isle of man or jamaica or jordan or kazakhstan or kazakh or kenya or "democratic people’s republic of korea" or republic of korea or north korea or south korea or korea or

kosovo or kyrgyzstan or kirghizia or kirgizstan or kyrgyz republic or kirghiz or laos or lao pdr or "lao people's democratic republic" or latvia or lebanon or lebanese republic or lesotho or basutoland or liberia or libya or libyan arab jamahiriya or lithuania or macau or macao or "macedonia (republic)" or macedonia or madagascar or malagasy republic or malawi or nyasaland or malaysia or malay federation or malaya federation or maldives or indian ocean islands or indian ocean or mali or malta or micronesia or federated states of micronesia or kiribati or marshall islands or nauru or northern mariana islands or palau or tuvalu or mauritania or mauritius or mexico or moldova or moldovian or mongolia or montenegro or morocco or ifni or mozambique or portuguese east africa or myanmar or burma or namibia or nepal or netherlands antilles or nicaragua or niger or nigeria or oman or muscat or pakistan or panama or papua new guinea or new guinea or paraguay or peru or philippines or philipines or phillipines or phillippines or poland or "polish people's republic" or portugal or portuguese republic or puerto rico or romania or russia or russian federation or ussr or soviet union or union of soviet socialist republics or rwanda or ruanda or samoa or pacific islands or polynesia or samoan islands or navigator island or navigator islands or "sao tome and principe" or saudi arabia or senegal or serbia or seychelles or sierra leone or slovakia or slovak republic or slovenia or melanesia or solomon island or solomon islands or norfolk island or norfolk islands or somalia or south africa or south sudan or sri lanka or ceylon or "saint kitts and nevis" or "st. kitts and nevis" or saint lucia or "st. lucia" or "saint vincent and the grenadines" or saint vincent or "st. vincent" or grenadines or sudan or suriname or surinam or dutch guiana or netherlands guiana or syria or syrian arab republic or tajikistan or tadjikistan or tadzhikistan or tadzhik or tanzania or tanganyika or thailand or siam or timor leste or east timor or togo or togolese republic or tonga or "trinidad and tobago" or trinidad or tobago or tunisia or turkey or "turkey (republic)" or turkmenistan or turkmen or uganda or ukraine or uruguay or uzbekistan or uzbek or vanuatu or new hebrides or venezuela or vietnam or viet nam or middle east or west bank or gaza or palestine or yemen or yugoslavia or zambia or zimbabwe or northern rhodesia or global south or africa south of the sahara or sub-saharan africa or subsaharan africa or africa, central or central africa or africa, northern or north africa or northern africa or magreb or maghrib or sahara or africa, southern or southern africa or africa, eastern or east africa or eastern africa or africa, western or west africa or western africa or west indies or indian ocean islands or caribbean or central america or latin america or "south and central america" or south america or asia, central or central asia or asia, northern or north asia or northern asia or asia, southeastern or southeastern asia or south eastern asia or southeast asia or south east asia or asia, western or westen asia or europe, eastern or east europe or eastern europe or developing country or developing countries or developing nation? or developing population? or developing world or less developed countr* or less developed nation? or less developed population? or less developed world or lesser developed countr* or lesser developed nation? or lesser developed population? or lesser developed world or under developed countr* or under developed nation? or under developed population? or under developed world or underdeveloped countr* or underdeveloped nation? or underdeveloped population? or underdeveloped world or middle income countr* or

middle income nation? or middle income population? or low income countr* or low income nation? or low income population? or lower income countr* or lower income nation? or lower income population? or underserved countr* or underserved nation? or underserved population? or underserved world or under served countr* or under served nation? or under served population? or under served world or deprived countr* or deprived nation? or deprived population? or deprived world or poor countr* or poor nation? or poor population? or poor world or poorer countr* or poorer nation? or poorer population? or poorer world or developing economy* or less developed economy* or lesser developed economy* or under developed economy* or underdeveloped economy* or middle income economy* or low income economy* or lower income economy* or low gdp or low gnp or low gross domestic or low gross national or lower gdp or lower gnp or lower gross domestic or lower gross national or lmic or lmics or third world or lami countr* or transitional countr* or emerging economies or emerging nation?)

6. TS=(afghan or afghans or afghani or albanian? algerian? or american samoan? or angolan? or antiguan? or barbudan? or argentine? or argentinian? or argentinean? or armenian? or aruban? or azerbaijani? or bahraini? or bangladeshi? or bangalees or bajan? or belarusian? or byelorussian? or belizean? or beninese? or bhutanese or bolivian? or bosnian? or botswana or batswana or brazilian? or brasilian? or bulgarian? or burkinabe or burkinese or burundian? or cape verdean? or cabo verdean? or cambodian? or khmer or cameroonian? or central african? or chadian? or chilean? or chinese or colombian? or comorian? or congolese or costa rican? or ivorian? or croatian? or cuban? or cypriot? or czech? or djiboutian? or dominican? or ecuadorian? or egyptian? or salvadoran? or equatorial guinean? or equatoguinean? or eritrean? or estonian? or swazi? or swati? or ethiopian? or fijian or gabonese or gabonaise or gambian? or georgian? or ghanaian? or gibraltarian? or greek? or grenadian? or guamanian? or guatemalan? or guinean? or bissau guinean? or guyanese or haitian? or honduran? or hungarian? or indian? or indonesian? or iranian? or iraqian? or iraqi? or manx or jamaican? or jordanian? or kazakhstani? or kenyan? or kirabati or kirabatian? or north korean? or korean? or kosovar? or kosovan? or kyrgyz* or lao or laotian? or latvian? or lebanese or lesothan? or lesothonian? or mosotho or basotho or liberian? or libyan? or lithuanian? or macanese or macedonian? or malagasy or madagascan? or malawian? or malaysian? or maldivian? or malian? or maltese or marshallese? or mauritanian? or mauritian? or mexican? or micronesian? or moldovan? or mongolian? or mongol or montenegrin? or moroccan? or mozambican? or burmese or myanma or namibian? or nauruan? or nepali or nepalese or netherlands antillean? or nicaraguan? or nigerien? or nigerian? or northern mariana islander? or mariana? or omani? or pakistani? or palauan? or panamanian? or papua new guinean? or paraguayan? or peruvian? or philippine? or philipine? or phillipine? or phillippine? or filipino? or filipina? or polish or pole or poles or portuguese or puerto rican? or romanian? or russian? or soviet people or soviet population or rwandan? or rwandese or ruandan? or ruandese or samoan? or sao tomean? or santomean? or saudi arabian? or saudi? or senegalese or serbian? or montenegrin? or seychellois or seychelloise? or sierra leonean? or slovak? or slovene? or solomon islander? or somali? or south african? or south sudanese or sri lankan? or ceylonese or kittitian? or nevisian?

or saint lucian? or vincentian? or sudanese or surinamese? or syrian? or tajik? or tajikistani? or tanzanian? or tanganyikan? or thai or timorese? or togolese or tongan? or trinidadian? or tobagonian? or tunisian? or turk? or turkish or turkmen? or tuvaluan? or ugandan? or ukrainian? or uruguayan? or uzbek? or vanuatu* or venezuelan? or vietnamese or yemeni? or yemenite? or yemenese or yugoslav? or yugoslavian? or zambian? or zimbabwean?)

7. #2 or #3
8. #5 or #6
9. #1 and #4 and #7 and #8
