# Supplementary material for: Parental participation in the care of hospitalized neonates in low- and middle-income countries: A systematic review and meta-analysis
Source: Front Pediatr. 2022 Aug 25;10:987228. doi: 10.3389/fped.2022.987228 (PMC9453204; doi:10.3389/fped.2022.987228)
Supplement: Supplementary file 2 [file Table_1.docx]

# Supplementary Table: Characteristics of Studies

| **Author and year** | **Design** | **Country** | **Number of infants** | **Population** | **Parental responsibilities in intervention group** | **Parental involvement in control group** | **Duration of intervention** |
| --- | --- | --- | --- | --- | --- | --- | --- |
| **Arif**  **1999 (11)** | Randomized controlled trial | Pakistan | 362 infants  (control 211; intervention 151) | Infants: 1-2kg on admission  Parents: Mothers | Mothers stayed at bedside  Mothers trained to do the following:   - Cleaning infant - Diaper changing - Feeding (including tube-feeding) - Monitor for apnea and gasping - Monitor incubator temperature | - Mothers stayed in room adjacent to the ward   - Mothers expressed breastmilk every 2 hours that was then fed to the infants | Duration of hospitalization |
| **Balbino 2016 (20)** | Pre-post cohort trial | Brazil | 98 infants (pre-intervention 47; post-intervention | Infants: Not specified  Parents: Mothers and fathers | Unit accessible to parents 24 hours per day. Parents involved in the following activities:   - Bathing infant - Decision-making - Diaper changing - Feeding (by glass or bottle) - Gavage feeding pre-discharge - Kangaroo care - Medication administration pre-discharge - Palliative care as needed - Tracheostomy and ostomy aspiration under nurse supervision | No parental involvement in care | Duration of hospitalization |
| **Bastani 2015 (12)** | Randomized controlled trial | Iran | 110  (control 55; intervention 55) | Infants: ≥2kg, gestational age 30-37 weeks, and diagnosed with respiratory distress syndrome. Congenital anomalies were excluded.  Parents: Mothers age 18-35 years, lack of chronic illness, literate, able to spend ≥ 18 hours/day with their baby | Mothers responsible for infants’ care after being taught about:   - Breathing exercises - Burping - Cause of their infant’s hospitalization - Changing infant’s position - Feeding methods (breastfeeding and gavage) - Frequency of feeds, bathing and handwashing and disinfectant solution - Infection control strategies - Leaving the unit at times - Milk storage   Pamphlets provided on:   - Skin-to-skin contact | No parental involvement in care | Duration of hospitalization |
| **Bhutta 2004 (23)** | Pre-post cohort trial | Pakistan | 509  (pre-intervention 191; post-intervention 318) | Infants: >2.5kg  Parents: Mothers | Mothers co-bedded with their infant. Mothers trained to:   - Feeding (breastfeeding, nasogastric tube, cup or spoon) - Monitor for danger signs and seek help as needed - Monitor temperature - Perform chest physiotherapy, suction and postural drainage - Wash hands before and after handling | Breastmilk feeding (from the breast, nasogastric tube, cup or spoon) | Duration of stay on the stepdown unit |
| **Djoeanda**  **1979 (13)** | Randomized controlled trial | Indonesia | 122  (control 62; intervention 60) | Infants: Not specified  Parents: Mothers | Mothers roomed-in with their babies. Mothers taught the following skills:   - Bathing of infant - Breastfeeding - Care of the umbilicus - Diaper changing | - Breastfeeding every three hours | Four days |
| **He**  **2018 (24)** | Pre-post cohort trial | China | 261  (pre-intervention 141; post-intervention 120) | Infants: Diagnosed with bronchopulmonary dysplasia (FiO2 ≥ 0.3 at 36 weeks and weight ≥ 1800 grams on ventilatory support or weight ≥ 1500 grams on non-invasive oxygen support). Neonates with severe congenital anomalies, surgery, receiving palliative care, and brain damage were excluded.  Parents: Mothers and fathers able to spend ≥ 3hours/day with their baby. Parents with social problems or language issues were excluded. | Parental presence at least three hours per day  Parents trained by nurses to do the following:   - Bathing - Diaper changing - Feeding (especially breastfeeding) - Hand hygiene - Other basic care Pat the infant’s back - Talk to their infant and play music - Touch the infant | No parental involvement  Parents updated by healthcare team by video three times per week | Duration of hospitalization |
| **Hei**  **2020 (14)** | Cluster randomized controlled trial | China | 601  (intervention 298; control 303) | Infants: Gestational age 28-35 weeks, receipt of enteral feeds >24 hours, vital signs stable > 24 hours. Ventilated infants, those receiving palliative care and those with surgical intervention were excluded.  Parents: Mothers and fathers able to spend >6 hours/day with their baby | Parents were trained to do the following:   - Bathing - Completion of patient’s daily chart (including ins and outs) - Diaper changing - Dressing the infant - Feeding (breastfeeding, gavage feeding)) - Hand hygiene - Monitoring of vitals - Participation in rounds - Positioning - Skin-to-skin contact - Skin, umbilical root and oral cleaning | Not reported | Duration of hospitalization |
| **Li**  **2017 (22)** | Retrospective cohort trial | China | 1446  (control 428; intervention 1018) | Infants: receiving oral feeds, not receiving supplemental oxygen, >1.5kg  Parents: Mothers, fathers or other relatives able to spend 24 hours/day with their baby | Parents trained by to do the following:   - Administering oral medications - Diaper changing - Feeding - Measuring the infant’s temperature - Participating in rounds and updating the infant’s information | No parental involvement  Parents updated by healthcare team twice a week, but can only enter the NICU with permission | Duration of hospitalization |
| **Lv 2019 (21)** | Non-randomized controlled trial | China | 319  (control 163; intervention 156) | Infants: Preterm with birthweight <1.5kg, non-invasive oxygen support. Infants with severe congenital anomalies, those who underwent surgery, and those receiving palliative care were excluded.  Parents: Mothers and fathers able to spend ≥4 hours/day with their baby | Parents trained to do the following:   - Bathing - Breastfeeding - Communication with infant - Diaper changing - Hand hygiene - Measuring the infant’s temperature - Skin-to-skin care | No parental involvement  Parents were updated by the healthcare team three times a week, and could only see their infant by video | Duration of hospitalization |
| **Moradi 2018 (15)** | Randomized controlled trial | Iran | 65  (control 32; intervention 33) | Infants: Gestational age 28-34 weeks, birthweight 1-2.5kg. Infants with congenital anomalies, major disease and neurological deficits were excluded.  Parents: Mothers age ≥18 years, junior high school education and above, no history of former infants’ hospitalization in NICU, absence of preeclampsia | Mothers received an empowerment program (bedside training, written material, and videos) that taught the following:  - Changing and bathing  - Hand hygiene   - Infection prevention - Medication administration - Milk preparation and maintenance - Recognizing danger signs and resuscitation - Sleep - Sucking optimization - Touching and massaging infant | Breastfeeding and skin-to-skin care | Duration of hospitalization |
| **Mirlashari 2021 (28)** | Non-randomized controlled trial | Iran | 80  (control 40; intervention 40) | Infants: Gestational age < 37 weeks; length of stay 1-4 weeks, infants with life-limiting disease were excluded  Parents: Fathers | Fathers received four 90-minute educational sessions that taught them to do the following:  - Feeding  - Developmental positioning  - Developmental handling  - Diaper changing  - Skin-to-skin care | Not reported | Four weeks |
| **Mustajab 1986 (25)** | Pre-post cohort trial | Indonesia | 649  (control 485; intervention 164) | Infants: Not reported  Parents: Mothers | Mothers roomed-in with infants 24 hours per day  Mothers were taught to do the following:   - Bathing infant - Breastfeeding, on demand - Diaper changing - Take care of the umbilicus | Not reported | Duration of hospitalization |
| **Namprom 2018 (16)** | Randomized controlled trial | Thailand | 50 (control 25; intervention 25) | Infants: Gestational age 28-32 weeks, birthweight < 2.5kg, singleton, inborn. Infants with a congenital anomaly were excluded  Parents: Mothers age ≥18 years, literate, no history of previous preterm infant in the NICU. | Maternal participation program (education plan, multi-touch interactive multimedia, and information handbook) on handling and caring for preterm infants, with hands-on training to do the following:   - Kangaroo mother care - Massage - Minimization of stress and pain - Optimization of nutrition - Oral care with colostrum - Positioning, handling - Promoting quality sleep environment and sleep - Protection of their skin - Provision of maternal scent (swabs soaked in breastmilk) - Recognition of and reaction to infant cues | Breastfeeding, provision of information of unit policies and resources to mothers | Birth to day 28 of life |
| **Namprom 2020 (18)** | Randomized controlled trial (follow-up to Namprom et al. 2018) | *Ibid.* | *Ibid.* | *Ibid.* | *Ibid.* | *Ibid.* | Day 28 of life to discharge |
| **Narayanan 1991 (29)** | Non-randomized controlled trial | India | 50  (control 25; intervention 25) | Infants: “high risk”  Parents: Mothers | Mothers roomed-in with their babies and provided the following care:   - Changed diapers - Cleaned the infant - Dressed the infant - Provided expressed breastmilk | No parental involvement. Mothers were asked to express breastmilk that was provided to the infant. | Duration of hospitalization |
| **Narayanan 1980 (26)** | Pre-post cohort trial | India | 179 infants  (control 72; intervention 107) | Infants: “high risk”, born outside of the hospital, “referred for special care.” Infants with sepsis were excluded.  Parents: Mother or other female caregiver | The caregivers roomed-in with their babies and were taught to do the following:   - Cleaned the infant - Diaper changing - Feeding the infant (breastmilk when available) - Hand hygiene | No parental involvement. | Duration of hospitalization |
| **Sasidharan 2005 (27)** | Pre-post cohort trial | India | 258 536 live births but number admitted to the neonatal unit not reported (control  31 031 live births; intervention 227 505 live births) | Infants: gestational age < 34 weeks or birthweight < 1.8kg or congenital malformation, or birth asphyxia or meconium aspiration.  Parents: Mother or “mother substitute” | Caregivers roomed-in with their babies and were taught to do the following:   - Aseptic methods for caring for their babies - Feeding (breastfeeding or feeding with expressed breast milk) - Hand hygiene - Recognizing danger signs | Mothers could visit and observe their babies. They could “occasionally” breastfeed. | Duration of hospitalization |
| **Verma 2017 (18)** | Randomized controlled trial | India | 295  (control 147; intervention 148) | Infants: Those with severe congenital anomalies, hemodynamically unstable, or the product of multiple gestation were excluded.  Parents: Mothers, fathers or other relatives | Caregivers were taught to do the following:   - Cleaning the infant - Covering the baby’s eyes and genitals during phototherapy - Feeding (breastfeeding, gavage, or spoon feeding) - Gowning - Hand hygiene - Monitoring pulse oximetry probe stability - Monitoring the warmer - Recognizing danger signs - Skin-to-skin care - Verifying IV cannula site | Skin-to-skin care and breastfeeding | Duration of hospitalization |
| **Zhang 2018 (19)** | Randomized controlled trial | China | 66 (control 33; intervention 33) | Infants: Gestational age < 37 weeks. Infants with severe congenital anomalies or on respiratory support were excluded.  Parents: Mothers and fathers who could spend ≥ 4 hours/day with their baby. Parents were excluded if they had “health family, social, or language issues”. | Parents learned about the following topics:  - Bathing the infant  - Feeding  - Hand hygiene  - Massage  - Neonatal resuscitation  - Respiratory support  Subsequently, parents participated as “primary caregivers” | Parents in the control group received the same education as parents in the intervention group. Parents in the control group were not allowed to visit their babies and were updated by the healthcare team three times per week. | Second week of hospitalization until discharge |
